# Clinicopathological and radiological significance of the collateral vessels of renal cell carcinoma on preoperative computed tomography

**DOI:** 10.1038/s41598-021-84631-w

**Published:** 2021-03-04

**Authors:** Xueling Suo, Junru Chen, Yijun Zhao, Qidun Tang, Xibiao Yang, Yuan Yuan, Ling Nie, Ni Chen, Hao Zeng, Jin Yao

**Affiliations:** 1 Department of Radiology, West China Hospital of Sichuan University, No. 37 Guo Xue Xiang, Chengdu, 610041 Sichuan China; 2 Department of Urology, Institute of Urology, West China Hospital of Sichuan University, No. 37 Guo Xue Xiang, Chengdu, 610041 Sichuan China; 3 Department of Radiology, The Second Xiangya Hospital of Central South University, Changsha, 410011 Hunan China; 4 Department of Urology, Chengdu Second People’s Hospital, Chengdu, 610017 Sichuan China; 5 Department of Pathology, West China Hospital of Sichuan University, Chengdu, 610041 Sichuan China

**Keywords:** Oncology, Urology

## Abstract

This study aimed to investigate the clinicopathological and radiological significance of the collateral vessel of renal cell carcinoma (RCC) on preoperative computed tomography (CT). Preoperative contrast-enhanced CT of 236 consecutive patients with pathological documented RCC were retrospectively reviewed during the period of 2014. The associations of the presence of collateral vessels with perioperative clinicopathological and radiological features, as well as long term survival outcomes were analyzed. Totally, collateral vessels were detected by contrast-enhanced CT in 110 of 236 patients. The presence of collateral vessels was significantly associated with higher pathologic T stage, higher Fuhrman grade, higher overall RENAL scores, greater tumor size and enhancement, and more tumor necrosis (all P < 0.05). In patients with clear cell RCC, those harboring collateral vessels had significantly higher SSIGN scores (P < 0.001) and shorter overall survival (P = 0.01) than those without collateral vessel. The incidence of intraoperative blood loss, blood transfusion, radical nephrectomy (RN) and open surgery were also significantly higher in patients with collateral vessels (all P < 0.05). In multivariate analysis, the presence of collateral vessels was significantly associated with RN (P = 0.021) and open surgery (P = 0.012). The presence of collateral vessels was significantly associated with aggressive clinicopathological parameters and worse prognosis. It is worth paying attention to its association with the choice of RN and open surgery in clinical practice.

## Introduction

Renal cell carcinoma (RCC) is one of the most common cancers in the genitourinary system, with estimated new cases and deaths of 403,262 and 175,098 in 2018, respectively^1^. Currently, surgery including radical nephrectomy (RN) and nephron-sparing surgery (NSS) is the mainstay treatment option for localized RCC, which can be performed via open or minimally invasive approaches. According to latest guidelines, T1 tumors are best managed by NSS, while RN is preferred for tumors ≥ T2^2^. However, many patients still experience perioperative complications and tumor recurrence^3,4^. Thus, accurate and comprehensive preoperative assessment is critical for the optimal management of RCC.

Contrast-enhanced computer tomography (CT) is widely used in the diagnosis, characterization and surveillance of RCC^5–7^. Imaging features such as tumor size, location, necrosis and tumor invasion into surrounding structures are commonly reported and regarded as important parameters by radiologists and surgeons^8–10^. However, several other imaging characteristics which are not routinely concerned and reported may also have potential value. The presence of collateral vessels on preoperative imaging is not uncommon in patients with RCC. One previous study has shown that patients with collateral vessels on CT scan was associated with greater tumor size and more aggressive histological subtypes of RCC^11^. Moreover, there are also studies indicating that the identification of collateral vessels could improve the accuracy of preoperative CT tumor staging and predict poor prognosis in patients with RCC^6,12^. Therefore, it is of great importance to have a comprehensive understanding of the associations of clinicopathological features as well as clinical outcomes with the presence of collateral vessels in CT images of RCC. In the present observative study, we aimed to explore the incidence of the presence of collateral vessels and investigate the clinicopathological and radiological significance of the collateral vessel in patients with RCC on preoperative CT scan.

## Results

### Associations between collateral vessels and clinicopathological characteristics after surgery

**Figure 1:**
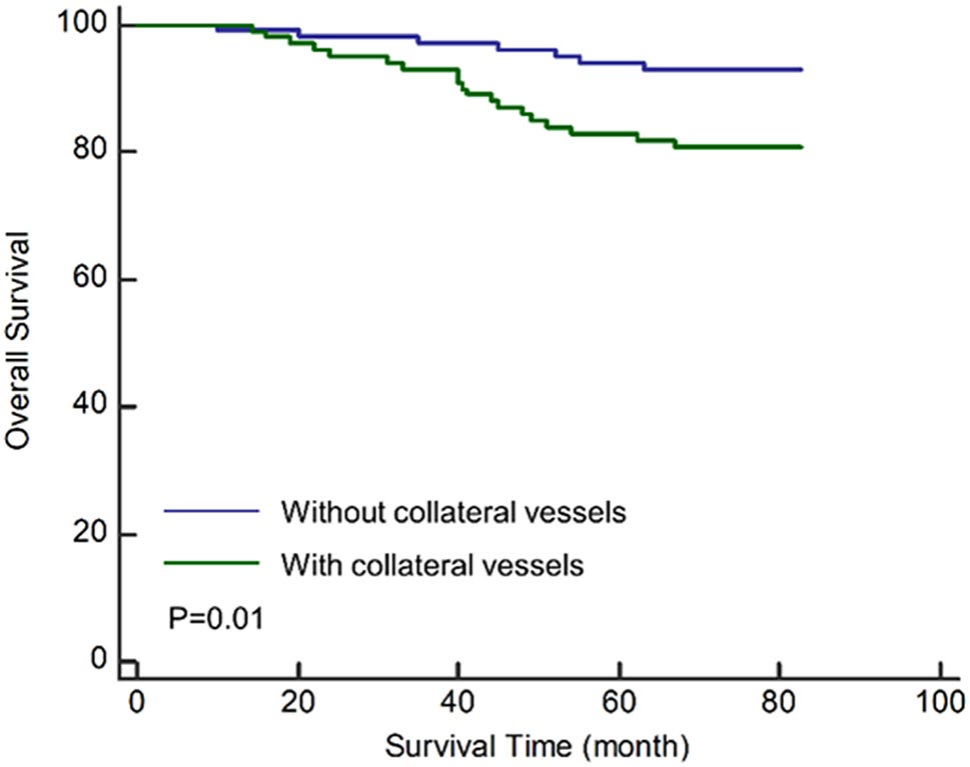
Kaplan–Meier curves of overall survival for patients with and without collateral vessels generated by MedCalc version 19.5.1 (MedCalc Software Ltd, Korea).

A total of 236 patients with RCC were included. The median time from CT scan to surgery was 9.8 days (range 0 to 64 days). The presence of collateral vessels was detected in 110/236 (44.6%) of patients by contrast-enhanced CT. The clinicopathological characteristics of the included patients were summarized in Table 1. Patients with collateral vessels had significantly higher pathologic tumor (pT) stage (P < 0.001), higher Fuhrman grade (P = 0.038) and higher incidence of necrosis (P = 0.003) compared to those without collateral vessels. Subgroup analyses indicated similar outcomes and showed that patients with collateral vessel diameter > 0.2 cm were associated with higher pT stage than those with collateral vessel diameter ≤ 0.2 cm (P = 0.004) (Supplementary Table S1-1,-2 online). Among 198 patients with clear cell RCC (ccRCC), those harboring collateral vessels had higher Stage, Size, Grade and Necrosis (SSIGN) scores than those without collateral vessels (Table 2, Supplementary Table S2-1,-2 online). Furthermore, survival analysis indicated that the presence of collateral vessels was significantly associated with poor overall survival (Fig. 1, 5 year survival rate: 81.9% vs. 94.1%, P = 0.01). After adjusting for other clinicopathological factors (age, gender, pT stage, Fuhrman grade and radiological necrosis), the presence of collateral vessels remained an independent predictor of overall survival (hazard ratio: 3.46; 95% CI 1.26–9.49; P = 0.016; Table 3).

**Table 1:** Clinicopathological characteristics of patients with RCC.

	RCC with collateral vessels (n = 110)	RCC without collateral vessels (n = 126)	P value
Age, mean ± SD, years	55.0 ± 13.1	55.0 ± 12.9	0.960
Gender (female)	43 (39.1%)	45 (35.7%)	0.686
**Pathologic T stage**			< 0.001
T1a	29 (26.4%)	78 (61.9%)	
T1b	42 (38.2%)	35 (27.8%)	
T2a	14 (12.7%)	7 (5.5%)	
T2b	6 (5.5%)	0 (0.0%)	
T3a	14 (12.7%)	3 (2.4%)	
T3b	3 (2.7%)	0 (0.0%)	
T4	2 (1.8%)	3 (2.4%)	
T stage upgrading	14 (12.7%)	18 (14.3%)	0.874
**Histologic patterns**			0.652
Clear cell	94 (85.4%)	104 (82.5%)	
Papillary	6 (5.5%)	11 (8.7%)	
Chromophobe	8 (7.3%)	7 (5.6%)	
Others	2 (1.8%)	4 (3.2%)	
**Fuhrman grading**			0.038
1–2	53 (48.2%)	75 (59.5%)	
3–4	48 (43.6%)	38 (30.2%)	
Undefined	9 (8.2%)	13 (10.3%)	
Necrosis	17 (15.5%)	5 (4.0%)	0.003
Perirenal fat invasion	7 (6.4%)	3 (2.4%)	0.117

*RCC* renal cell carcinoma.

**Table 2:** SSIGN scores in ccRCC patients with and without collateral vessels.

	ccRCC with collateral vessels (n = 94)	ccRCC without collateral vessels (n = 104)	P value
SSIGN score ≤ 2	52 (55.3%)	91 (87.5%)	< 0.001
SSIGN score > 2	42 (44.7%)	13 (12.5%)	

*ccRCC* clear cell renal cell carcinoma; *SSIGN* stage, size, grade and necrosis.

**Table 3:** Univariate and multivariate analyses of factors in predicting overall survival.

	Overall survival			
	Univariate analysis		Multivariate analysis	
	HR (95%Cl)	P value	HR (95%Cl)	P value
**Age, years**				
≥ Median vs < median	1.05 (0.48–2.27)	0.906		
**Gender**				
Male vs Female	0.96 (0.45–2.05)	0.914		
**Radiological necrosis**				
Yes vs No	1.18 (0.52–2.69)	0.689		
**Collateral vessels**				
Yes vs No	3.20 (1.36–7.54)	**0.008**	3.46 (1.26–9.49)	**0.016**
**Pathologic T stage**				
> 2 vs ≤ 2	3.98 (1.80–8.81)	**0.001**	2.92 (1.23–6.94)	**0.015**
Fuhrman grading				
> 2 vs ≤ 2	3.01 (1.26–7.22)	**0.013**		

*HR* hazard ratio; *CI* confidence interval.

### Associations between collateral vessels and other imaging features on preoperative CT

The imaging features on CT of patients with and without collateral vessels were summarized in Table 4. Tumor size of patients with collateral vessels was significantly larger than those without collateral vessels (5.9 ± 2.4 cm vs. 3.8 ± 1.7 cm, P < 0.001). The presence of calcification (18.2% vs. 8.7%, P = 0.035), necrosis (80.9% vs. 55.6%, P = 0.001), perirenal fat invasion (21.8% vs. 7.1%, P = 0.001) and renal vein invasion (10.9% vs. 2.4%, P = 0.013) on CT were more common in patients with collateral vessels compared to those without collateral vessels. No significant differences in tumor location, renal vein thrombus, or renal sinus invasion were observed between the two groups. The mean attenuation values (142.6 ± 57.0 vs. 119.1 ± 56.8 Hounsfield Unit (HU), P < 0.001) and tumor-to-renal contrast (-13.2 ± 49.4 vs. -29.1 ± 49.0 HU, P = 0.018) during corticomedullary phase (CMP) were significantly different in patients with and without collateral vessels. The mean attenuation value or tumor-to-renal contrast during the unenhanced phase and nephrographic phase (NP) did not differ significantly between these two populations. Subgroup analyses revealed similar results and further indicated that patients with collateral vessel diameter > 0.2 cm were associated with more aggressive imaging features than those with collateral vessel diameter ≤ 0.2 cm (Supplementary Table S3-1,-2 online).

**Table 4:** Imaging characteristics of RCC with and without collateral vessels.

	RCC with collateral vessels (n = 110)	RCC without collateral vessels (n = 126)	P value
Tumor size (cm)	5.9 ± 2.4	3.8 ± 1.7	< 0.001
**Tumor location**			0.696
Left	59 (53.6%)	64 (50.8%)	
Right	51 (46.4%)	62 (49.2%)	
Necrosis	89 (80.9%)	70 (55.6%)	0.001
Calcification	20 (18.2%)	11 (8.7%)	0.035
Perirenal fat invasion	24 (21.8%)	9 (7.1%)	0.001
Renal vein invasion	12 (10.9%)	3 (2.4%)	0.013
Renal vein thrombus	5 (4.5%)	2 (1.6%)	0.256
Renal sinus invasion	18 (16.4%)	11 (8.7%)	0.111
**Tumor attenuation, HU**			
Unenhanced	35.9 ± 6.6	35.1 ± 8.5	0.363
Corticomedullary phase	142.6 ± 57.0	119.1 ± 56.8	< 0.001
Nephrographic phase	131.2 ± 42.6	123.8 ± 46.1	0.133
**Tumor to renal contrast, HU**			
Unenhanced	4.3 ± 6.8	3.3 ± 9.2	0.202
Corticomedullary phase	− 13.2 ± 49.4	− 29.1 ± 49.0	0.018
Nephrographic phase	− 45.6 ± 39.2	− 51.2 ± 41.8	0.391

*RCC* renal cell carcinoma; *HU* Hounsfield Unit.

Among 110 patients with collateral vessels, 10 cases manifested as collateral arteries, 67 cases with collateral veins, and 33 cases with both collateral arteries and veins. The most common sources of collateral arteries were renal artery, adrenal artery and abdominal aorta. For the collateral veins, the common sources included gonadal, renal, adrenal, intercostal and lumbar veins and inferior vena cava (Supplementary Table S3-3 online).

### Associations between collateral vessel and perioperative parameters

The perioperative parameters were compared between patients with and without collateral vessels (Table 5). Patients with collateral vessels had significantly higher overall RENAL scores (median score: 9 vs. 7, P < 0.001), more blood loss (275.4 ± 534.4 vs. 125.3 ± 231.9 mL, P = 0.037) and higher incidence of blood transfusion (6.4% vs. 0.8%, P = 0.02) during operation than those without collateral vessels. The percentages of RN (78.2% vs. 51.6%, P < 0.001) and open surgery (61.8% vs. 36.5%, P < 0.001) were significantly higher in patients with collateral vessels. The differences in operating time and hospital stay were not statistically significant between these two groups. Subgroup analyses showed that patients with collateral vessel diameter > 0.2 cm were associated with higher RENAL scores, longer operation time, more proportion of RN, more intraoperative blood transfusion and longer hospital stay than those with collateral vessel diameter ≤ 0.2 cm (Supplementary Table S4-1,-2 online).

**Table 5:** Perioperative parameters of RCC patients with and without collateral vessels.

	Overall	RCC with collateral vessels (*n* = 110)	RCC without collateral vessels (*n* = 126)	P value
RENAL score, median (range)	8(4 ~ 12)	9(4 ~ 12)	7(4 ~ 12)	< 0.001
Operating time (min)	131.0 ± 33.8	133.5 ± 38.7	128.7 ± 28.8	0.721
**Operation type**				< 0.001
NSS	85(36.0%)	24(21.8%)	61(48.4%)	
RN	151(64.0%)	86(78.2%)	65(51.6%)	
**Operation approach**				< 0.001
Laparoscopic surgery	122(51.7%)	42(38.2%)	80(63.5%)	
Open surgery	114(48.3%)	68(61.8%)	46(36.5%)	
Blood loss, mean ± SD, ml	190.3 ± 398.4	275.4 ± 534.4	125.3 ± 231.9	0.037
Intraoperative blood transfusion	8(3.4%)	7(6.4%)	1(0.8%)	0.020
Hospital stay (days)	11.3 ± 3.9	11.9 ± 4.2	10.9 ± 3.5	0.050

*RCC* renal cell carcinoma; *NSS* nephron-sparing surgery; *RN* radical nephrectomy.

As shown in Table 6, in multivariate analysis, the presence of collateral vessels was independently associated with RN (OR 2.32, 95% CI 1.13–4.73, P = 0.021) and open surgery (OR 2.06, 95% CI 1.17–3.61, P = 0.012). However, there was no significant association of RN and open surgery with different collateral vessel type, collateral vessel amount and collateral vessel diameter (Table 7).

**Table 6:** Univariate and multivariate analyses of different clinicopathological factors in predicting radical nephrectomy and open surgery.

	Radical nephrectomy				Open surgery			
	Univariate analyses		Multivariate analyses		Univariate analyses		Multivariate analyses	
	OR (95% CI)	p value	OR (95% CI)	P value	OR (95% CI)	p value	OR (95% CI)	P value
**Gender **								
Male vs. female	0.88 (0.50–1.52)	0.635			1.54 (0.90–2.62)	0.114		
**Age**								
> 50 vs. ≤ 50 years	0.96 (0.55–1.68)	0.958			1.45 (0.85–2.49)	0.176		
**RENAL score**								
7–9 vs.4–6	6.53 (3.26–13.09)	< 0.001	5.98 (2.88–12.44)	< 0.001	0.92 (0.49–1.71)	0.793	0.74 (0.38–1.41)	0.355
10–12 vs. 4–6	200.00 (25.65–1559.61)	< 0.001	100.92 (12.47–816.64)	< 0.001	2.33 (1.15–4.71)	0.018	0.970 (0.42–2.26)	0.944
**cT stage**								
> T1 vs. ≤ T1	18.53 (5.60–61.33)	< 0.001	4.86 (1.32–17.89)	0.017	4.23 (2.24–7.97)	< 0.001	3.28 (1.56–6.93)	0.002
Collateral vessels with vs. without	3.36 (1.90–5.96)	< 0.001	2.32 (1.13–4.73)	0.021	2.72 (1.61–4.61)	< 0.001	2.06 (1.17–3.61)	0.012

**Table 7:** Univariate and multivariate analyses of different subgroups of collateral vessels in predicting radical nephrectomy and open surgery.

	Radical nephrectomy				Open surgery			
	Univariate analyses		Multivariate analyses*		Univariate analyses		Multivariate analyses*	
	OR (95% CI)	p value	OR (95% CI)	P value	OR (95% CI)	p value	OR (95% CI)	P value
**Collateral vessels type**								
Artery vs. none	4.11 (1.77–9.55)	0.001	3.11 (1.06–9.12)	0.039	3.88 (1.84–8.17)	< 0.001	3.09 (1.43–6.67)	0.004
Vein vs. none	2.99 (1.54–5.80)	0.001	2.06 (0.92–4.62)	0.078	2.20 (1.21–4.03)	0.01	1.91 (1.03–3.55)	0.041
Artery vs. vein	0.73 (0.28–1.89)	0.514	0.66 (0.21–2.10)	0.476	0.57 (0.25–1.28)	0.171	0.66 (0.28–1.60)	0.36
**Number of collateral vessels**								
1 vs. 0	2.87 (1.53–5.35)	0.001	2.32 (1.08–5.00)	0.032	2.36 (1.32–4.30)	0.004	2.11 (1.17–3.81)	0.014
2 vs. 0	5.26 (1.91–14.48)	0.001	2.53 (0.71–9.01)	0.152	3.87 (1.70–8.83)	0.001	2.81 (1.18–6.67)	0.019
2 vs. 1	1.83 (0.62–5.42)	0.272	1.21 (0.33–4.40)	0.772	1.64 (0.69 = 3.90)	0.268	1.19 (0.45–3.11)	0.731
**Collateral vessels diameter**								
≤ 0.2 cm vs.0 cm	2.32 (1.23–4.39)	0.01	2.20 (1.01–4.73)	0.046	2.28 (1.24–4.19)	0.008	2.20 (1.38–4.07)	0.013
> 0.2 cm vs. 0 cm	7.32 (2.71–19.79)	< 0.001	3.05 (0.87–10.69)	0.082	3.60 (1.74–7.47)	0.001	2.43 (1.18–5.34)	0.027
> 0.2 cm vs. ≤ 0.2 cm	3.15 (1.08–9.22)	0.036	1.54 (0.43–5.49)	0.509	1.58 (0.71–3.52)	0.263	0.79 (0.30–2.06)	0.627

*The multivariate analyses were conducted including parameters with p value < 0.05 in univariate analyses in Table 6.

## Discussion

To the best of our knowledge, this was the first study to comprehensively investigate the clinicopathological and radiological significance of collateral vessels on CT in patients with pathological documented RCC. In this study, we found that the presence of collateral vessel was not uncommon (as high as 44.6%) and it was significantly associated with greater tumor size and more aggressive histopathological characteristics. Furthermore, patients with collateral vessels were also found to have higher RENAL scores and more adverse perioperative events. Patients with collateral vessels might be more prone to RN and open surgery. Furthermore, the presence of collateral vessels may help predict long-term prognosis of patients with RCC.

The imaging features of RCC are various. Tumor size, tumor invasion and necrosis have been claimed to be associated with tumor staging and prognosis^6,10,13^. Thus, identification of imaging characteristics to guide clinical practice and predict patient outcomes is of great importance. RCC is a hypervascular tumor due to high constitutive production of vascular endothelial growth factor activated by hypoxia-inducible factor^14^. Growing evidence has shown that high levels of angiogenesis was associated with poor prognosis in RCC^15,16^. Currently, microvessel density is frequently used to assess intratumoral angiogenesis via immunohistochemical staining. Collateral vessels on preoperative imaging is not uncommon in RCC and its role is not well established. To date, just few studies have investigated the presence of collateral vessels of kidney tumor on preoperative imaging.

A study from the Memorial Sloan-Kettering Cancer Center reported that the presence of peritumoral vascularity was significantly associated with tumor size within each subtype of RCC^11^, which aligned with our results. Similarly, a previous angiography study demonstrated that Wilms' tumors with collateral vessels had a relatively larger size than those without collaterals^17^. In line with our findings, Bradley and colleagues retrospectively reviewed 92 patients with RCC and demonstrated that the presence of collateral vessels was a reliable sign of locally advanced renal cancer^6^. In addition, another study from the USA demonstrated that the presence of collateral vessels on MRI before surgery was an independent predictor of high-grade clear cell type^18^.

Collateral veins were more frequently observed than collateral arteries in the present study, which might be explained by lower blood pressure of venous system. Collateral veins were present in the forms of the gonadal, renal, adrenal, intercostal, and lumbar veins, and inferior vena cava in our study, which was concordant with previous publications^19–21^. It was worth noting that collateral arteries and veins often coexisted. A renal tumor developing in the peripheral renal parenchyma close to a perforating artery may act as a stimulation for the perirenal arterial plexus, and partial blood flow of the tumor located in this region may return through the perirenal venous complex^22^. Thus, collateral arteries and veins could coexist in some cases. In the present study, the incidence of necrosis was higher in tumors with collateral vessels which might have faster growth exceeding the blood supply and finally leading to necrosis.

Surgical resection remains the standard treatment of localized RCC. However, debate persists on the optimal surgery modality for patients with RCC, especially early stage RCC. RN is effective but may result in potentially higher possibility of renal dysfunction^23^. NSS may provide similar oncological outcomes and better renal function but is probably associated with technical complexity^24^. Therefore, patient physical status, comorbidity, surgeon experience and surgical complexity should be considered in decision making. RENAL scoring system stratifies renal masses into low, intermediate, and high complexity^9^, providing implications for surgical planning. The present study found that patients with collateral vessels were significantly associated with higher RENAL scores, which indicated the presence of collateral vessels on preoperative CT could be considered as a cofactor to help estimate the complexity of surgery. Satasivam and colleagues reported that renal masses dissected by RN were predominantly with moderate to high complexity, whereas NSS was used mainly for low-complexity lesions^25^. Rosevear et al. also found that patients treated with RN had higher RENAL scores than those received NSS^26^. According to our results, the presence of collateral vessels was an independent predictor of RN as well as open surgery. In addition, increased blood loss and higher rate of intraoperative blood transfusion in patients with collateral vessels in our study might also be explained by the higher tumor complexity and increased collateral circulation. Thus, under a trend of increasing use of NSS and minimally invasive surgery^27^, these findings might supply a clue that RN and open surgery should have priority in patients with collateral vessels because of higher surgical complexity, and if minimally invasive surgery is still chosen, surgeons should at least be consistently alert to higher possibility of bleeding during operation. Moreover, our results showed that patients with collateral vessels had higher SSIGN scores and worse overall survival than those without collateral vessels, which indicated that the presence of collateral vessels might be a predictor of prognosis and help guide clinicians make decisions for additional subsequent treatments.

It was also worth noting that this study had some limitations. First, the study was retrospective and limited by selection bias. Second, only CMP and NP images were acquired, and the findings (e.g. urinary tract invasion by the tumor) in the excretory phase could not be analyzed, which will need to be addressed in future studies. Third, tumor size was the main concern of tumor staging, especially for tumor stage < T3. The association between the presence of collateral vessels and tumor size might influence the predictive power of collateral vessels for tumor stage and other clinicopathological features. Fourth, previous studies have shown that imaging-based definition of tumor necrosis sometimes does not demonstrate necrosis at histologic evaluation, which may represent focal fibrosis, colloid or glycogen in the cells, or focal cystic change^28^. That may contribute to the mismatch between the incidence of necrosis evaluated by CT and by pathological examination.

## Conclusions

The presence of collateral vessels was significantly associated with aggressive clinicopathological characteristics of RCC tumor lesion and poor survival outcomes. It was related to higher surgical complexity and more perioperative complications. The presence of collateral vessels was an independent predictor for potential RN and open surgery. Therefore, fully evaluation of collateral vessels in preoperative CT may have clinical potential in management of patients with RCC.

## Materials and methods

### Patient enrollment and clinicopathological data

A retrospective review of the electronic medical record system at our hospital between January 2014 and December 2014 was conducted. The inclusion criteria were: (1) patients with resection of pathologically confirmed RCC; (2) patients underwent contrast-enhanced CT or CT angiography of the abdomen covering the whole kidney and the perirenal space within 3 months before surgery. Patients with history of surgery in ipsilateral kidney for other reasons or with vascular diseases were excluded. The clinicopathological data, including age, gender, pT stage, histologic pattern, tumor Fuhrman grading, and other pathological-related characteristics were collected. At the same time, perioperative data, including surgical approach, surgery type, operation time, blood loss, intraoperative blood transfusion and hospital stay were also collected. Histopathological diagnosis was reviewed by two genitourinary pathologists, according to 2016 World Health Organization classification^29^. The R.E.N.A.L. and SSIGN scores were calculated according to criteria reported in previous studies^9,30^. Ethical approval for this study was obtained from Institution Review Board of West China Hospital of Sichuan University, and written informed consent was obtained from all participants. The methods were performed in accordance with the approved guidelines.

### Imaging techniques

CT examinations were performed using a 64-detector row system (Brilliance 64; Philips Medical Systems, Eindhoven, Netherlands) and a dual source system (Somatom Definition AS/FLASH; Siemens Healthcare, Forchheim, Germany) in a single tube mode. Detailed CT acquisition parameters are shown in Supplementary Table S5 online.

For patients who underwent a routine abdominal enhanced CT (n = 197), after an initial unenhanced CT scan, contrast agent (Iohexol, 300 mg iodine/mL; Bayer Schering Pharma AG, Leverkusen, Germany) dosed to weight (1.0–1.5 mL/kg) was injected with an automatic power injector (Stellant D Dual Syringe, Medrad, Indianola, PA, USA) at a rate of 2.5–3.0 mL/s, the CMP and NP began around 30–35 s and 60–70 s after contrast injection; for patients who underwent an abdominal CT angiography (n = 39), the injection rate was 4.0–5.0 mL/s and an automatic triggering system was used to time the start of scanning of the arterial and venous phase^31^. A region of interest (ROI) cursor (1.0–2.0 cm^2^) was placed in the abdominal aorta at the level of the celiac axis, with a trigger set to begin at 100 or 120 HU. The arterial and venous phase images were acquired within 25–30 s and 40–45 s after injection of the contrast agent.

### Image analysis

All CT images were reviewed independently by two radiologists (X.S. and Y.Z. with 4 years of experience in abdominal imaging diagnosis). Any discrepancies were resolved by discussion with another well-experienced abdominal radiologist to make a consensus (J.Y. with 18 years of experience in abdominal imaging diagnosis). A picture archiving and communication system (Syngo; Siemens Medical Systems, Forchheim, Germany) was used to evaluate the CT images by adjusting the optimal window setting in each case. Three-dimensional soft-tissue reconstruction algorithm was applied to generate coronal and sagittal images to aid the interpretation.

**Figure 2:**
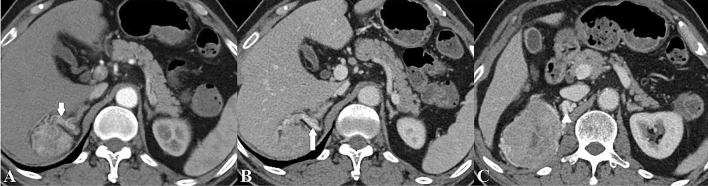
A 70-year-old man with a 7.3 cm clear cell carcinoma in the right kidney. Contrast-enhanced computed tomographic images demonstrate a prominent peritumoral collateral artery (short arrow) during the corticomedullary phase (**A**) and collateral vein (long arrow) during the nephrographic phase (**B**) in the right perinephric space. The collateral vein during the nephrographic phase (**C**) measures 0.5 cm in diameter (arrowhead). The collateral artery originates from the right adrenal artery, and the collateral vein is present in the form of inferior vena cava.

**Figure 3:**
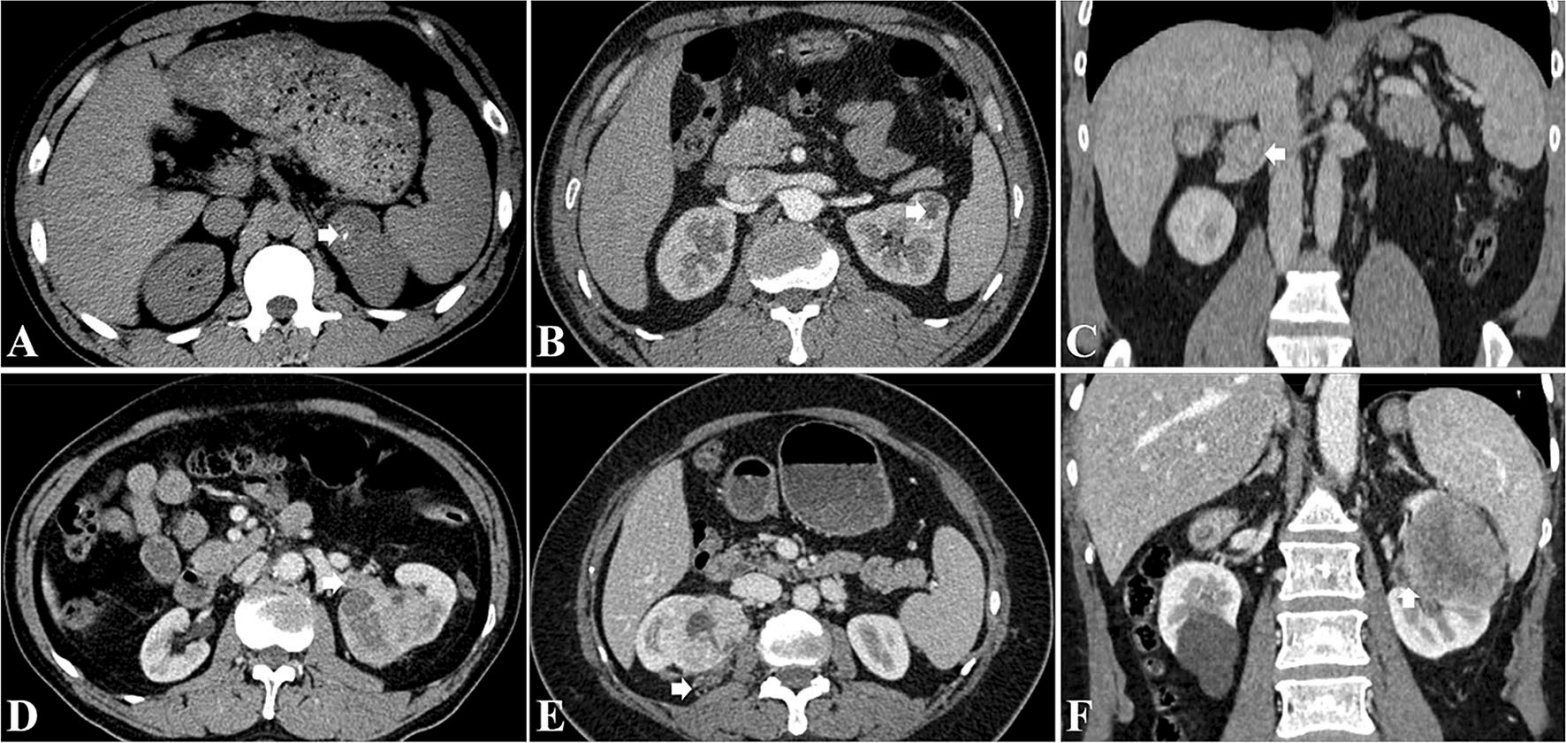
CT imaging features as assessed by both readers: (**A**) calcification; (**B**) necrosis; (**C**) renal vein invasion; (**D**) renal vein thrombus; (**E**), perirenal fat invasion; (**F**) renal sinus invasion.

The presence of collateral vessel was defined as asymmetrically increased, usually irregular vessel within Gerota’s fascia or renal hilum of the kidney^11^, collateral arteries and veins were identified on the CMP and NP images, respectively (Fig. 2). The following imaging features were recorded: tumor size (maximal diameter), tumor location, the presence of calcification, necrosis, perirenal fat invasion, renal sinus invasion, renal vein invasion and thrombus (Fig. 3)^8,32^. Tumor attenuation and the tumor-to-renal contrast were quantitatively assessed. For heterogeneous lesions, a round or elliptic ROI was placed in the area that had the greatest degree of enhancement of the renal tumor in the CMP and NP; for homogeneous lesions, ROIs were placed in the center of the renal tumor^33^. The ROIs measured approximately 0.2–1 cm^2^ and were consistent in size and location on the unenhanced phase, CMP and NP images^32^. ROIs were also placed in the adjacent normal renal cortex for normalization^33^. At least three measurements were performed for each lesion and renal cortex on three CT scans, with averaged results^34^. The tumor-to-renal contrast was measured by subtracting the tumor attenuation from the normal renal cortex attenuation during the unenhanced phase, CMP and NP, respectively.

### Statistical analysis

Statistical analyses were performed using SPSS version 24.0 (SPSS, Chicago, IL, USA) and MedCalc version 19.5.1 (MedCalc Software Ltd, Korea). Statistical differences among clinicopathological, perioperative data, and CT imaging features were analyzed using Chi-square test or Fisher’s exact test for categorical variables, Student t test or Mann–Whitney U test for continuous variables. Survival curves were generated by the Kaplan–Meier method and compared by log-rank test. Subgroup analyses based on characteristics of collateral vessels were conducted. Multivariate logistic regression analyses were performed to determine independent factors predicting the risk of RN and open surgery. Cox proportional hazards regression (forward likelihood ratio model) was used to determine independent predictor of overall survival. A P value of less than 0.05 was considered to denote statistical significance.

## Supplementary Information

Supplementary Information.

## Data Availability

The datasets generated during and/or analyzed during the current study are available from the corresponding author on reasonable request.
