# Supplementary material for: Clinicopathological and radiological significance of the collateral vessels of renal cell carcinoma on preoperative computed tomography
Source: Sci Rep. 2021 Mar 4;11:5187. doi: 10.1038/s41598-021-84631-w (PMC7933355; doi:10.1038/s41598-021-84631-w)
Supplement: Supplementary file 1 — Supplementary Information. [file 41598_2021_84631_MOESM1_ESM.docx]

**Supplementary materials**

**Clinicopathological and radiological significance of the collateral vessels of renal cell carcinoma on preoperative computed tomography**

Xueling Suo^1^, Junru Chen^2^, Yijun Zhao^3^, Qidun Tang^4^, Xibiao Yang^1^, Yuan Yuan^1^, Ling Nie^5^, Ni Chen^5^, Hao Zeng^2*^, and Jin Yao^1*^

^1^ Department of Radiology, West China Hospital of Sichuan University, Chengdu, Sichuan 610041, China

^2^ Department of Urology, Institute of Urology, West China Hospital of Sichuan University, Chengdu, Sichuan 610041, China

^3^ Department of Radiology, the Second Xiangya Hospital of Central South University, Changsha, Hunan 410011, China

^4^ Department of Urology, Chengdu Second People’s Hospital, Chengdu, Sichuan 610017, China

^5^ Department of Pathology, West China Hospital of Sichuan University, Chengdu, Sichuan 610041, China

Xueling Suo and Junru Chen contributed to this work equally.

***Correspondence to**: Hao Zeng and Jin Yao

**Address**: Department of Urology, Institute of Urology, West China Hospital, Sichuan University, No. 37 Guo Xue Xiang, Chengdu, Sichuan 610041, China; Department of Radiology, West China Hospital, Sichuan University, No. 37 Guo Xue Xiang, Chengdu, Sichuan 610041, China.

**E-mail**: [kucaizeng@163.com](mailto:kucaizeng@163.com) and [shelleyyao@163.com](mailto:shelleyyao@163.com); **Tel**: 086-028 85423130; **Fax**: 086-028 8542274

**Supplementary Table S1-1**. Subgroup analyses of clinicopathological characteristics in patients with and without collateral vessels.

|  | Without collateral vessels (n=126) | Collateral artery  (n=43) | p value | Collateral vein  (n=67) | p  value | Number of collateral vessels=1  (n=77) | p  value | Number of collateral vessels=2  (n=33) | p  value | Diameter of collateral vessels≤0.2cm (n=66) | p value | Diameter of collateral vessels>0.2cm (n=44) | p value |
| --- | --- | --- | --- | --- | --- | --- | --- | --- | --- | --- | --- | --- | --- |
| Age, mean ± SD, years | 55.0±12.9 | 57.7$\pm$12.3 | 0.232 | 53.4$\pm$13.4 | 0.420 | 54.1$\pm$13.3 | 0.634 | 57.3$\pm$12.5 | 0.343 | 52.8$\pm$14.1 | 0.480 | 58.4$\pm$10.8 | 0.112 |
| Gender | 45 (35.7%) | 16 (37.2%) | 0.860 | 27 (40.3%) | 0.531 | 28 (36.4%) | 0.952 | 15 (45.5%) | 0.304 | 27 (40.9%) |  | 16 (36.4%) | 0.938 |
| Pathologic  T stage |  |  | <0.001 |  | <0.001 |  | <0.001 |  | <0.001 |  | 0.008 |  | <0.001 |
| T1a | 78 (61.9%) | 7 (16.3%) |  | 22 (32.8%) |  | 26 (33.8%) |  | 3 (9.1%) |  | 24 (36.4%) |  | 5 (11.4%) |  |
| T1b | 35 (27.8%) | 17 (39.5%) |  | 25 (37.3%) |  | 28 (36.4%) |  | 14 (42.4%) |  | 28 (42.4%) |  | 14 (31.8%) |  |
| T2a | 7 (5.5%) | 7 (16.3%) |  | 6 (9.0%) |  | 7 (9.1%) |  | 6 (18.2%) |  | 6 (9.1%) |  | 7 (15.9%) |  |
| T2b | 0 (0.0%) | 3 (7.0%) |  | 3 (4.5%) |  | 4 (5.2%) |  | 2 (6.1%) |  | 2 (3.0%) |  | 4 (9.1%) |  |
| T3a | 3 (2.4%) | 7 (16.3%) |  | 8 (11.9%) |  | 9 (11.7%) |  | 6 (18.2%) |  | 5 (7.6%) |  | 10 (22.7%) |  |
| T3b | 0 (0.0%) | 1 (2.3%) |  | 2 (3.0%) |  | 2 (2.6%) |  | 1 (3.0%) |  | 0 (0%) |  | 3 (6.8%) |  |
| T4 | 3 (2.4%) | 1 (2.3%) |  | 1 (1.5%) |  | 1 (1.3%) |  | 1 (3.0%) |  | 1 (1.5%) |  | 1 (2.3%) |  |
| Histologic patterns |  |  | 0.961 |  | 0.503 |  | 0.740 |  | 0.711 |  | 0.605 |  | 0.626 |
| Clear cell | 104 (82.5%) | 37 (86.0%) |  | 57 (85.1%) |  | 65 (84.4%) |  | 29 (87.9%) |  | 55 (83.3%) |  | 39 (88.6%) |  |
| Papillary | 11 (8.7%) | 3 (7.0%) |  | 3 (4.5%) |  | 4 (5.2%) |  | 2 (6.1%) |  | 3 (4.5%) |  | 3 (6.8%) |  |
| Chromophobe | 7 (5.6%) | 2 (4.7%) |  | 6 (9.0%) |  | 6 (7.8%) |  | 2 (6.1%) |  | 6 (9.1%) |  | 2 (4.5%) |  |
| Others | 4 (3.2%) | 1 (2.3%) |  | 1 (1.5%) |  | 2 (2.6%) |  | 0 (0%) |  | 2 (3.0%) |  | 0 (0%) |  |
| Fuhrman grading |  |  | 0.035 |  | <0.001 |  | 0.001 |  | 0.013 |  | 0.003 |  | 0.002 |
| 1-2 | 75 (59.5%) | 19 (44.2%) |  | 23 (34.3%) |  | 29 (37.7%) |  | 13 (39.4%) |  | 25 (37.9%) |  | 17 (38.6%) |  |
| 3-4 | 38 (30.2%) | 21 (48.8%) |  | 38 (56.7%) |  | 41 (53.2%) |  | 18 (54.5%) |  | 33 (50.0%) |  | 26 (59.1%) |  |
| Undefined | 13 (10.3%) | 3 (7.0%) |  | 6 (9.0%) |  | 7 (9.1%) |  | 2 (6.1%) |  | 8 (12.1%) |  | 1 (2.3%) |  |
| Necrosis | 5 (4.0%) | 11 (25.6%) | <0.001 | 6 (9.0%) | 0.155 | 7 (9.1%) | 0.218 | 10 (30.3%) | <0.001 | 8 (12.1%) | 0.067 | 9 (20.5%) | 0.002 |
| Perirenal fat invasion | 3 (2.4%) | 5 (11.6%) | 0.014 | 1 (1.5%) | 0.680 | 2 (2.6%) | 1.000 | 4 (12.1%) | 0.051 | 2 (3.0%) | 1.000 | 4 (9.1%) | 0.137 |

**Supplementary Table S1-2**. Subgroup analyses of clinicopathological characteristics in patients with collateral vessels.

|  | Collateral artery  (n=43) | Collateral vein  (n=67) | p value | Number of collateral vessels=1  (n=77) | Number of collateral vessels=2 (n=33) | p value | Diameter of collateral vessels≤0.2cm (n=66) | Diameter of collateral vessels>0.2cm (n=44) | p value |
| --- | --- | --- | --- | --- | --- | --- | --- | --- | --- |
| Age, mean ± SD, years | 57.7$\pm$12.3 | 53.4$\pm$13.4 | 0.093 | 54.1$\pm$13.3 | 57.3$\pm$12.5 | 0.230 | 52.8$\pm$14.1 | 58.4$\pm$10.8 | 0.027 |
| Gender | 16 (37.2%) | 27 (40.3%) | 0.746 | 28 (36.4%) | 15 (45.5%) | 0.371 | 27 (40.9%) | 16 (36.4%) | 0.632 |
| Pathologic T stage |  |  | 0.576 |  |  | 0.216 |  |  | 0.004 |
| T1a | 7 (16.3%) | 22 (32.8%) |  | 26 (33.8%) | 3 (9.1%) |  | 24 (36.4%) | 5 (11.4%) |  |
| T1b | 17 (39.5%) | 25 (37.3%) |  | 28 (36.4%) | 14 (42.4%) |  | 28 (42.4%) | 14 (31.8%) |  |
| T2a | 7 (16.3%) | 6 (9.0%) |  | 7 (9.1%) | 6 (18.2%) |  | 6 (9.1%) | 7 (15.9%) |  |
| T2b | 3 (7.0%) | 3 (4.5%) |  | 4 (5.2%) | 2 (6.1%) |  | 2 (3.0%) | 4 (9.1%) |  |
| T3a | 7 (16.3%) | 8 (11.9%) |  | 9 (11.7%) | 6 (18.2%) |  | 5 (7.6%) | 10 (22.7%) |  |
| T3b | 1 (2.3%) | 2 (3.0%) |  | 2 (2.6%) | 1 (3.0%) |  | 0 (0%) | 3 (6.8%) |  |
| T4 | 1 (2.3%) | 1 (1.5%) |  | 1 (1.3%) | 1 (3.0%) |  | 1 (1.5%) | 1 (2.3%) |  |
| T stage upgrading | 6 (14.0%) | 8 (11.9%) | 0.987 | 10 (13.0%) | 4 (12.1%) | 0.851 | 12 (18.2%) | 2 (4.5%) | 0.070 |
| Histologic patterns |  |  | 0.784 |  |  | 0.797 |  |  | 0.490 |
| Clear cell | 37 (86.0%) | 57 (85.1%) |  | 65 (84.4%) | 29 (87.9%) |  | 55 (83.3%) | 39 (88.6%) |  |
| Papillary | 3 (7.0%) | 3 (4.5%) |  | 4 (5.2%) | 2 (6.1%) |  | 3 (4.5%) | 3 (6.8%) |  |
| Chromophobe | 2 (4.7%) | 6 (9.0%) |  | 6 (7.8%) | 2 (6.1%) |  | 6 (9.1%) | 2 (4.5%) |  |
| Others | 1 (2.3%) | 1 (1.5%) |  | 2 (2.6%) | 0 (0%) |  | 2 (3.0%) | 0 (0%) |  |
| Fuhrman grading |  |  | 0.329 |  |  | 0.962 |  |  | 0.719 |
| 1-2 | 19 (44.2%) | 23 (34.3%) |  | 29 (37.7%) | 13 (39.4%) |  | 25 (37.9%) | 17 (38.6%) |  |
| 3-4 | 21 (48.8%) | 38 (56.7%) |  | 41 (53.2%) | 18 (54.5%) |  | 33 (50.0%) | 26 (59.1%) |  |
| Undefined | 3 (7.0%) | 6 (9.0%) |  | 7 (9.1%) | 2 (6.1%) |  | 8 (12.1%) | 1 (2.3%) |  |
| Necrosis | 11 (25.6%) | 6 (9.0%) | 0.019 | 7 (9.1%) | 10 (30.3%) | 0.005 | 8 (12.1%) | 9 (20.5%) | 0.236 |
| Perirenal fat invasion | 5 (11.6%) | 1 (1.5%) | 0.022 | 2 (2.6%) | 4 (12.1%) | 0.119 | 2 (3.0%) | 4 (9.1%) | 0.346 |

**Supplementary Table S2-1**. Subgroup analyses of SSIGN scores in clear cell renal cell carcinoma patients with and without collateral vessels.

|  | Without collateral vessels (n=104) | Collateral artery  (n=37) | p value | Collateral vein  (n=57) | p value | Number of collateral vessels=1  (n=65) | p value | Number of collateral vessels=2 (n=29) | p value | Diameter of collateral vessels≤0.2cm (n=55) | p value | Diameter of collateral vessels>0.2cm (n=39) | p value |
| --- | --- | --- | --- | --- | --- | --- | --- | --- | --- | --- | --- | --- | --- |
| SSIGN score ≤2 | 91 (87.5%) | 15 (40.5%) | <0.001 | 37 (64.9%) | 0.001 | 43 (66.2%) | 0.001 | 9 (31.0%) | <0.001 | 38 (69.1%) | 0.005 | 14 (35.9%) | <0.001 |
| SSIGN score >2 | 13 (12.5%) | 22 (59.5%) |  | 20 (35.1%) |  | 22 (33.8%) |  | 20 (69.0%) |  | 17 (30.9%) |  | 25 (64.1%) |  |

SSIGN, Stage, Size, Grade and Necrosis.

**Supplementary Table S2-2**. Subgroup analyses of SSIGN scores in clear cell renal cell carcinoma patients with collateral vessels.

|  | Collateral artery  (n=37) | Collateral vein  (n=57) | p value | Number of collateral vessels=1  (n=65) | Number of collateral vessels=2  (n=29) | p value | Diameter of collateral vessels≤0.2cm (n=55) | Diameter of collateral vessels>0.2cm (n=39) | p value |
| --- | --- | --- | --- | --- | --- | --- | --- | --- | --- |
| SSIGN score ≤2 | 15 (40.5%) | 37 (64.9%) | 0.020 | 43 (66.2%) | 9 (31.0%) | 0.002 | 38 (69.1%) | 14 (35.9%) | 0.001 |
| SSIGN score >2 | 22 (59.5%) | 20 (35.1%) |  | 22 (33.8%) | 20 (69.0%) |  | 17 (30.9%) | 25 (64.1%) |  |

SSIGN, Stage, Size, Grade and Necrosis.

**Supplementary Table S3-1**. Subgroup analyses of imaging characteristics in patients with and without collateral vessels.

|  | Without collateral vessels (n=126) | Collateral artery  (n=43) | p value | Collateral vein  (n=67) | p value | Number of collateral vessels=1  (n=77) | p value | Number of collateral vessels=2 (n=33) | p value | Diameter of collateral vessels≤0.2cm (n=66) | p value | Diameter of collateral vessels>0.2cm (n=44) | p value |
| --- | --- | --- | --- | --- | --- | --- | --- | --- | --- | --- | --- | --- | --- |
| Tumor size (cm) | 3.8±1.7 | 6.5$\pm$2.4 | <0.001 | 5.6$\pm$2.4 | <0.001 | 5.6$\pm$2.6 | <0.001 | 6.7$\pm$1.8 | <0.001 | 5.2$\pm$2.3 | <0.001 | 7.1$\pm$2.2 | <0.001 |
| Tumor location |  |  | 0.967 |  | 0.677 |  | 0.734 |  | 0.701 |  | 0.761 |  | 0.185 |
| Left | 64 (50.8%) | 21 (48.8%) |  | 37 (55.2%) |  | 41 (53.2%) |  | 18 (54.5%) |  | 32 (48.5%) |  | 27 (61.4%) |  |
| Right | 62 (49.2%) | 22 (51.2%) |  | 30 (44.8%) |  | 36 (46.8%) |  | 15 (45.5%) |  | 34 (51.5%) |  | 17 (38.6%) |  |
| Necrosis | 70 (55.6%) | 38 (88.4%) | <0.001 | 48 (71.6%) | 0.038 | 56 (72.7%) | 0.008 | 29 (87.9%) | 0.001 | 50 (75.8%) | 0.006 | 36 (81.8%) | 0.451 |
| Calcification | 11 (8.7%) | 9 (20.9%) | 0.032 | 11 (16.4%) | 0.549 | 11 (14.3%) | 0.217 | 9 (27.3%) | 0.008 | 6 (9.1%) | 0.933 | 14 (31.8%) | 0.002 |
| Perirenal fat invasion | 9 (7.1%) | 13 (30.2%) | <0.001 | 11 (16.4%) | 0.087 | 13 (16.9%) | 0.030 | 11 (33.3%) | <0.001 | 9 (13.6%) | 0.143 | 15 (34.1%) | 0.011 |
| Renal vein invasion | 3 (2.4%) | 5 (11.6%) | 0.026 | 7 (10.4%) | 0.846 | 7 (9.1%) | 0.044 | 5 (15.2%) | 0.010 | 1 (1.5%) | 1.000 | 11 (25.0%) | <0.001 |
| Renal vein thrombus | 2 (1.6%) | 1 (2.3%) | 1.000 | 4 (6.0%) | 0.371 | 4 (5.2%) | 0.203 | 1 (3.0%) | 0.505 | 0 (0%) | 0.546 | 5 (11.4%) | 0.019 |
| Renal sinus invasion | 11 (8.7%) | 7 (16.3%) | 0.249 | 11 (16.4%) | 0.985 | 11 (14.3%) | 0.217 | 7 (21.2%) | 0.062 | 2 (3.0%) | 0.225 | 16 (36.4%) | <0.001 |
| Tumor attenuation, HU |  |  |  |  |  |  |  |  |  |  |  |  |  |
| Unenhanced | 35.1±8.5 | 36.0$\pm$6.0 | 0.447 | 35.9$\pm$6.9 | 0.508 | 35.9$\pm$7.0 | 0.393 | 35.6$\pm$5.6 | 0.680 | 35.3$\pm$7.1 | 0.884 | 36.9$\pm$5.7 | 0.110 |
| Corticomedullary phase | 119.1±56.8 | 153.1$\pm$74.1 | 0.008 | 135.8$\pm$41.9 | 0.036 | 134.7$\pm$43.8 | 0.043 | 161.5$\pm$78.1 | 0.006 | 142.5$\pm$56.7 | 0.007 | 142.6$\pm$58.2 | 0.020 |
| Nephrographic phase | 123.8±46.1 | 134.5$\pm$49.8 | 0.201 | 129.1$\pm$37.5 | 0.523 | 129.8$\pm$44.0 | 0.338 | 133.9$\pm$40.6 | 0.254 | 132.6$\pm$48.5 | 0.220 | 129.2$\pm$32.1 | 0.476 |
| Tumor to renal contrast, HU |  |  |  |  |  |  |  |  |  |  |  |  |  |
| Unenhanced | 3.3±9.2 | 4.1$\pm$6.5 | 0.510 | 4.4$\pm$7.0 | 0.346 | 4.6$\pm$7.1 | 0.202 | 3.2$\pm$6.0 | 0.965 | 3.8$\pm$6.6 | 0.654 | 5.0$\pm$7.0 | 0.249 |
| Corticomedullary phase | -29.1±49.0 | -11.3$\pm$53.3 | 0.055 | -14.5$\pm$47.1 | 0.057 | -16.0$\pm$48.8 | 0.076 | -5.6$\pm$51.8 | 0.017 | -16.0$\pm$47.8 | 0.077 | -9.1$\pm$52.0 | 0.023 |
| Nephrographic phase | -51.2±41.8 | -47.1$\pm$32.8 | 0.515 | -44.7$\pm$43.0 | 0.311 | -44.7$\pm$42.1 | 0.304 | -47.2$\pm$33.5 | 0.615 | -42.6$\pm$37.0 | 0.163 | -50.2$\pm$42.4 | 0.890 |

**Supplementary Table S3-2**. Subgroup analyses of imaging characteristics in patients with collateral vessels.

|  | Collateral artery  (n=43) | Collateral vein  (n=67) | p value | Number of collateral vessels=1  (n=77) | Number of collateral vessels=2  (n=33) | p value | Diameter of collateral vessels≤0.2cm (n=66) | Diameter of collateral vessels>0.2cm (n=44) | p value |
| --- | --- | --- | --- | --- | --- | --- | --- | --- | --- |
| Tumor size (cm) | 6.5$\pm$2.4 | 5.6$\pm$2.4 | 0.053 | 5.6$\pm$2.6 | 6.7$\pm$1.8 | 0.014 | 5.2$\pm$2.3 | 7.1$\pm$2.2 | <0.001 |
| Tumor location |  |  | 0.677 |  |  | 0.900 |  |  | 0.185 |
| Left | 21 (48.8%) | 37 (55.2%) |  | 41 (53.2%) | 18 (54.5%) |  | 32 (48.5%) | 27 (61.4%) |  |
| Right | 22 (51.2%) | 30 (44.8%) |  | 36 (46.8%) | 15 (45.5%) |  | 34 (51.5%) | 17 (38.6%) |  |
| Necrosis | 38 (88.4%) | 48 (71.6%) | 0.038 | 56 (72.7%) | 29 (87.9%) | 0.107 | 50 (75.8%) | 36 (81.8%) | 0.451 |
| Calcification | 9 (20.9%) | 11 (16.4%) | 0.549 | 11 (14.3%) | 9 (27.3%) | 0.106 | 6 (9.1%) | 14 (31.8%) | 0.002 |
| Perirenal fat invasion | 13 (30.2%) | 11 (16.4%) | 0.087 | 13 (16.9%) | 11 (33.3%) | 0.056 | 9 (13.6%) | 15 (34.1%) | 0.011 |
| Renal vein invasion | 5 (11.6%) | 7 (10.4%) | 0.846 | 7 (9.1%) | 5 (15.2%) | 0.350 | 1 (1.5%) | 11 (25.0%) | <0.001 |
| Renal vein thrombus | 1 (2.3%) | 4 (6.0%) | 0.371 | 4 (5.2%) | 1 (3.0%) | 0.617 | 0 (0%) | 5 (11.4%) | 0.019 |
| Renal sinus invasion | 7 (16.3%) | 11 (16.4%) | 0.985 | 11 (14.3%) | 7 (21.2%) | 0.368 | 2 (3.0%) | 16 (36.4%) | <0.001 |
| Tumor attenuation, HU |  |  |  |  |  |  |  |  |  |
| Unenhanced | 36.0$\pm$6.0 | 35.9$\pm$6.9 | 0.935 | 35.9$\pm$7.0 | 35.6$\pm$5.6 | 0.731 | 35.3$\pm$7.1 | 36.9$\pm$5.7 | 0.195 |
| Corticomedullary phase | 153.1$\pm$74.1 | 135.8$\pm$41.9 | 0.167 | 134.7$\pm$43.8 | 161.5$\pm$78.1 | 0.069 | 142.5$\pm$56.7 | 142.6$\pm$58.2 | 0.998 |
| Nephrographic phase | 134.5$\pm$49.8 | 129.1$\pm$37.5 | 0.523 | 129.8$\pm$44.0 | 133.9$\pm$40.6 | 0.669 | 132.6$\pm$48.5 | 129.2$\pm$32.1 | 0.682 |
| Tumor to renal contrast, HU |  |  |  |  |  |  |  |  |  |
| Unenhanced | 4.1$\pm$6.5 | 4.4$\pm$7.0 | 0.838 | 4.6$\pm$7.1 | 3.2$\pm$6.0 | 0.280 | 3.8$\pm$6.6 | 5.0$\pm$7.0 | 0.351 |
| Corticomedullary phase | -11.3$\pm$53.3 | -14.5$\pm$47.1 | 0.743 | -16.0$\pm$48.8 | -5.6$\pm$51.8 | 0.290 | -16.0$\pm$47.8 | -9.1$\pm$52.0 | 0.477 |
| Nephrographic phase | -47.1$\pm$32.8 | -44.7$\pm$43.0 | 0.755 | -44.7$\pm$42.1 | -47.2$\pm$33.5 | 0.783 | -42.6$\pm$37.0 | -50.2$\pm$42.4 | 0.325 |

**Supplementary Table S3-3.** The sources of collateral vessels in RCC.

| Collateral vessels | Number | Collateral arteries | Number | Collateral veins | Number |
| --- | --- | --- | --- | --- | --- |
| Collateral arteries | 10 | Renal artery | 25 | Gonadal vein | 48 |
| Collateral veins | 67 | Abdominal aorta | 10 | Renal vein | 46 |
| Arteries and veins | 33 | Adrenal artery | 8 | Inferior vena cava | 17 |
|  |  | Iliac artery | 2 | Adrenal vein | 12 |
|  |  | Hepatic artery | 1 | Intercostal vein | 11 |
|  |  | Splenic artery | 1 | Lumbar vein | 5 |
|  |  | Celiac trunk | 1 | Splenic vein | 2 |
|  |  |  |  | Iliac vein | 1 |
|  |  |  |  | Inferior phrenic veins | 1 |

RCC, renal cell carcinoma

**Supplementary Table S4-1**. Subgroup analyses of perioperative parameters in patients with and without collateral vessels.

|  | Without collateral vessels (n=126) | Collateral artery  (n=43) | p value | Collateral vein  (n=67) | p value | Number of collateral vessels=1  (n=77) | p value | Number of collateral vessels=2  (n=33) | p value | Diameter of collateral vessels≤0.2cm (n=66) | p value | Diameter of collateral vessels>0.2cm (n=44) | p value |
| --- | --- | --- | --- | --- | --- | --- | --- | --- | --- | --- | --- | --- | --- |
| RENAL score, median (range) | 7 (4~12) | 9 (4-12) | 0.001 | 9 (4-12) | 0.003 | 9 (4-12) | 0.007 | 10 (5-12) | <0.001 | 8 (4-11) | 0.076 | 10 (4-12) | <0.001 |
| Operating time (min) | 128.7±28.8 | 136.6$\pm$41.6 | 0.273 | 130.9$\pm$37.3 | 0.727 | 131.0$\pm$36.3 | 0.686 | 138.0$\pm$44.6 | 0.279 | 117.0$\pm$24.8 | 0.010 | 157.4$\pm$43.7 | <0.001 |
| Operation type |  |  | 0.001 |  | 0.001 |  | 0.001 |  | 0.001 |  | 0.009 |  | <0.001 |
| NSS | 61 (48.4%) | 8 (18.6%) |  | 16 (23.9%) |  | 19 (24.7%) |  | 5 (15.2%) |  | 19 (28.8%) |  | 5 (11.4%) |  |
| RN | 65 (51.6%) | 35 (81.4%) |  | 51 (76.1%) |  | 58 (75.3%) |  | 28 (84.8%) |  | 47 (71.2%) |  | 39 (88.6%) |  |
| Operation approach |  |  | <0.001 |  | 0.010 |  | 0.003 |  | 0.001 |  | 0.007 |  | <0.001 |
| Laparoscopic surgery | 80 (63.5%) | 13 (30.2%) |  | 28 (41.8%) |  | 32 (41.6%) |  | 10 (30.3%) |  | 28 (42.4%) |  | 14 (31.8%) |  |
| Open surgery | 46 (36.5%) | 30 (69.8%) |  | 39 (58.2%) |  | 45 (58.4%) |  | 23 (69.7%) |  | 38 (57.6%) |  | 30 (68.2%) |  |
| Blood loss, mean ± SD, ml | 125.3±231.9 | 182.3$\pm$279.8 | 0.249 | 327.0$\pm$629.6 | 0.088 | 197.0±306.1 | 0.204 | 303.6$\pm$595.1 | 0.119 | 173.3$\pm$235.8 | 0.235 | 427.1$\pm$774.2 | 0.022 |
| Intraoperative blood transfusion | 1 (0.8%) | 2 (4.7%) | 0.324 | 5 (7.5%) | 0.035 | 5 (6.5%) | 0.057 | 2 (6.1%) | 0.207 | 0 (0%) | 1.000 | 7 (15.9%) | <0.001 |
| Hospital stay (days) | 10.9±3.5 | 11.9$\pm$3.4 | 0.196 | 11.5$\pm$3.6 | 0.187 | 11.6$\pm$3.5 | 0.155 | 11.9$\pm$3.6 | 0.263 | 10.9$\pm$3.0 | 0.718 | 12.9$\pm$4.0 | 0.002 |

NSS, nephron-sparing surgery; RN, radical nephrectomy.

|  | Collateral artery  (n=43) | Collateral vein  (n=67) | p value | Number of collateral vessels=1  (n=77) | Number of collateral vessels=2  (n=33) | p value | Diameter of collateral vessels≤0.2cm (n=66) | Diameter of collateral vessels>0.2cm (n=44) | p  value |
| --- | --- | --- | --- | --- | --- | --- | --- | --- | --- |
| RENAL score, median (range) | 9 (4-12) | 9 (4-12) | 0.509 | 9 (4-12) | 10 (5-12) | 0.069 | 8 (4-11) | 10 (4-12) | 0.001 |
| Operating time (min) | 136.6$\pm$41.6 | 130.9$\pm$37.3 | 0.454 | 131.0$\pm$36.3 | 138.0$\pm$44.6 | 0.390 | 117.0$\pm$24.8 | 157.4$\pm$43.7 | <0.001 |
| Operation type |  |  | 0.513 |  |  | 0.268 |  |  | 0.030 |
| NSS | 8 (18.6%) | 16 (23.9%) |  | 19 (24.7%) | 5 (15.2%) |  | 19 (28.8%) | 5 (11.4%) |  |
| RN | 35 (81.4%) | 51 (76.1%) |  | 58 (75.3%) | 28 (84.8%) |  | 47 (71.2%) | 39 (88.6%) |  |
| Operation approach |  |  | 0.327 |  |  | 0.328 |  |  | 0.062 |
| Laparoscopic surgery | 13 (30.2%) | 28 (41.8%) |  | 32 (41.6%) | 10 (30.3%) |  | 28 (42.4%) | 14 (31.8%) |  |
| Open surgery | 30 (69.8%) | 39 (58.2%) |  | 45 (58.4%) | 23 (69.7%) |  | 38 (57.6%) | 30 (68.2%) |  |
| Blood loss, mean ± SD, ml | 182.3$\pm$279.8 | 327.0±629.6 | 0.229 | 197.0$\pm$306.1 | 303.6$\pm$595.1 | 0.610 | 173.3$\pm$235.8 | 427.1$\pm$774.2 | 0.178 |
| Intraoperative blood transfusion | 2 (4.7%) | 5 (7.5%) | 0.850 | 5 (6.5%) | 2 (6.1%) | 1.000 | 0 (0%) | 7 (15.9%) | 0.003 |
| Hospital stay (days) | 11.9$\pm$3.4 | 11.5$\pm$3.6 | 0.581 | 11.6$\pm$3.5 | 11.9$\pm$3.6 | 0.682 | 10.9$\pm$3.0 | 12.9$\pm$4.0 | 0.003 |

**Supplementary Table S4-2**. Subgroup analyses of perioperative parameters in patients with collateral vessels.

NSS, nephron-sparing surgery; RN, radical nephrectomy.

**Supplementary Table S5.** CT imaging protocol parameters.

| Parameter | 64-detector row scanner | Dual source scanner |
| --- | --- | --- |
| Tube voltage (kV) | 120 | 120 |
| Tube current (mAs) | 200 | 210 |
| Rotation time (s) | 0.62 | 0.5 |
| Pitch | 0.891 | 0.8 |
| Detector collimation (mm) | 64 × 0.625 | 128 × 0.6 |
| Section thickness (mm) | 1-2 | 1-2 |
| Section interval (mm) | 1-2 | 1-2 |
